# Optoelectronic performance of dye-sensitized solar cells sensitized with chlorophyll-rich extracts from *Physalis angulata* L. (mullaca): laboratory and stratospheric evaluation

**DOI:** 10.1039/d6ra09523b

**Published:** 2026-11-05

**Authors:** Angie Pierina Perez Jara, Michael Celestino Cajavilca, Clemente Alfredo Luyo Caycho, Harry Anderson Rivera Tito, Yanett del Pilar Huanca Villanes, Jaime Claros Castellares, María Esther Quintana Caceda

**Affiliations:** a Center for the Development of Advanced Materials and Nanotechnology, National University of Engineering Av. Tupac Amaru 210 Lima 25 Peru mquintana@uni.edu.pe; b National University of Central Peru, Faculty of Chemical Engineering Peru jclaros@uncp.edu

## Abstract

*Physalis angulata* L. (mullaca), a plant rich in chlorophyll, was investigated as a natural sensitizer for dye-sensitized solar cells (DSSCs) to promote sustainable photovoltaic technologies. Nanostructured TiO_2_ photoelectrodes were sensitized using ethanolic extracts of mullaca prepared under various extraction conditions to evaluate their influence on photovoltaic performance. The DSSCs were characterized using optical, structural, and photovoltaic techniques under simulated solar illumination. Furthermore, two mullaca sensitized devices (C5 and C6) were evaluated during a stratospheric flight *via* the “Astrotracker” payload, aboard NASA's High Altitude Student Platform (HASP). The best-performing device obtained in the laboratory (C3: leaves, 48-hour immersion) achieved a maximum power conversion efficiency of 0.21%, with an open-circuit voltage of approximately 0.64 V. The four top-performing laboratory devices (C1–C4) exhibited fill factors ranging from 0.64 to 0.71 at 25 °C and 1 atm. UV-vis spectra revealed absorption bands characteristic of chlorophyll-rich pigments, supporting their role in visible-light harvesting. During the stratospheric experiment, the two devices that underwent the flight maintained a measurable electrical response under conditions of low pressure and variable natural illumination. Data from the cells were recorded during the flight, and electrical parameters (*V*_OC_, raw *I*_SC_, *P*_max_ and FF) were derived accordingly. These findings indicate that *Physalis angulata* can serve as a natural sensitizer for DSSCs and provide preliminary evidence of its electrical functionality under stratospheric flight conditions.

## Abstract 2

Chlorophyll-rich extracts from *Physalis angulata* sensitized TiO_2_ solar cells, yielding 0.21% laboratory efficiency. Stratospheric tests provided preliminary, uncalibrated electrical measurements.

## Graphical abstract

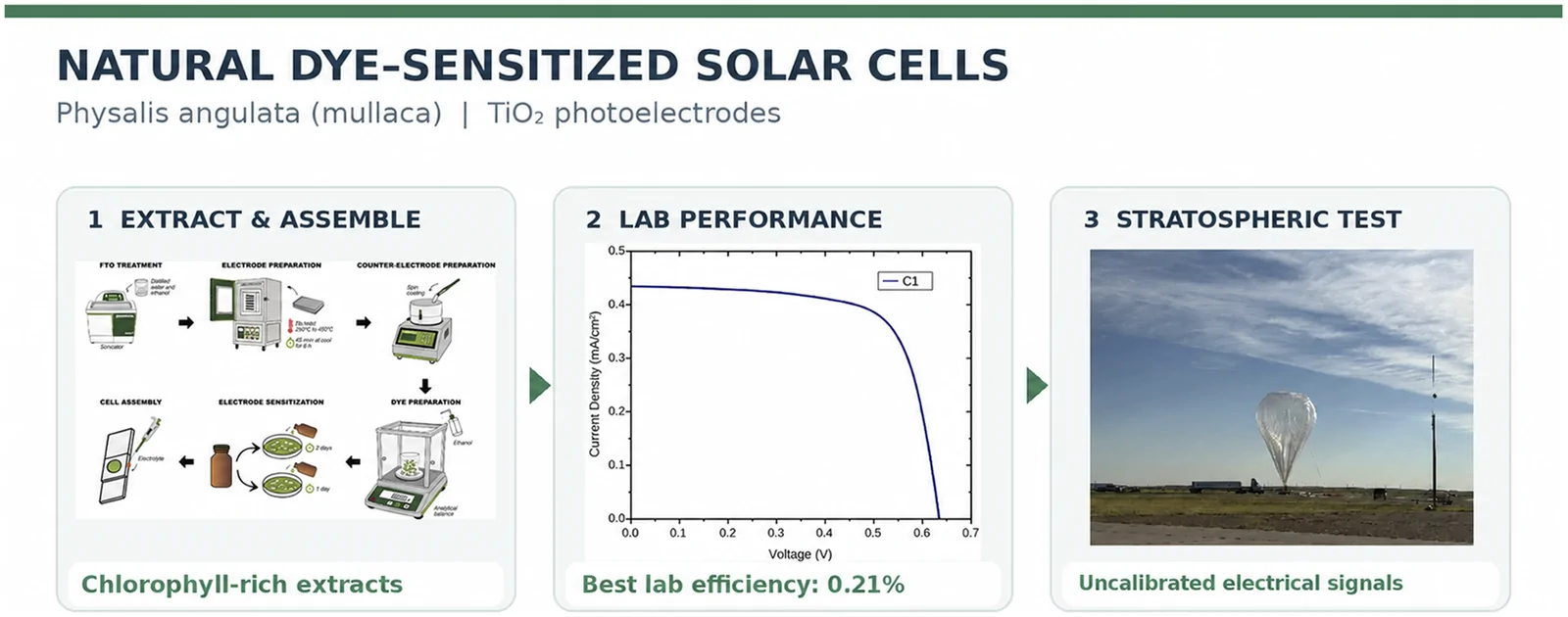

## Introduction

Photovoltaic technologies include thin-film CIGS and CdTe cells^1–3^ and emerging perovskite devices.^4^ Dye-sensitized solar cells (DSSCs) are third-generation photovoltaic devices^5^ composed of a nanostructured wide-bandgap semiconductor, typically titanium dioxide (TiO_2_), a redox electrolyte, and a molecular sensitizer that absorbs sunlight and injects electrons into the semiconductor conduction band.^6,7^ TiO_2_ is widely used because of its chemical stability and wide band gap.^8,9^ Among these components, the sensitizer plays a pivotal role because it governs light harvesting, charge separation, and electron recombination, thereby strongly influencing the overall photovoltaic performance.^10,11^

To date, the highest power conversion efficiencies, typically exceeding 10% under standard AM 1.5 G illumination, have been achieved using ruthenium-based sensitizers such as the N3 dye.^12–14^ Despite their excellent performance, these dyes are expensive, rely on relatively scarce metals, and raise environmental concerns associated with their synthesis and disposal.^15^ Consequently, considerable efforts have been devoted to developing sustainable, low-cost alternatives based on naturally occurring pigments.^8,16–21^

In this context, natural dyes extracted from leaves, flowers, fruits, roots, and seeds have emerged as environmentally friendly and low-cost alternatives to conventional metal-complex sensitizers.^22,23^ Chlorophylls, anthocyanins, carotenoids, and betalains have been extensively investigated as photosensitizers in DSSCs, generally yielding power conversion efficiencies between 0.1 and 1.5%.^11,17,22^ Although these values remain below those obtained with ruthenium complexes, natural dyes offer important advantages in terms of sustainability, abundance, and environmental compatibility.^11,17,23^ Efficiency improvements in DSSCs have also been pursued through photoanode engineering, such as metal-ion doping, composite oxides and alternative oxides (ZnO, SnO_2_).^24–28^

Beyond photovoltaics, plant leaf extracts and dyes attract broad interest in fields such as the chemical characterization and bioactivity of leaf extracts,^29^ fluorescent sensing and bioimaging,^30^ colorimetric sensing on textiles^31^ and light-driven photosynthetic systems,^32^ while power-electronic systems and electrical drives are likewise central to modern energy conversion.^33^

Peru possesses exceptional biodiversity together with high solar irradiance, making it an attractive setting for the development of natural-dye-based DSSCs.^34^ Numerous native plant species remain unexplored as potential photosensitizers despite containing pigments with suitable optical properties.^35–37^ Among these species is *Physalis angulata* L., locally known as mullaca, a plant traditionally valued for its medicinal and nutritional properties but scarcely studied as a source of photosensitizing pigments.^38^ Previous studies have reported that mullaca contains pigments and other compounds with absorption bands in the visible region, suggesting that its extract may act as an effective dye for TiO_2_ photoelectrodes, consistent with observations in other systems.^23,38,39^

In this work, the use of chlorophyll-rich extracts obtained from *Physalis angulata* L. leaves as a natural sensitizer in TiO_2_ based DSSCs is specifically explored. Evaluating natural sensitizers under extreme environmental conditions is also of growing interest for future aerospace and high-altitude photovoltaic applications, where devices are exposed to low pressure, large temperature fluctuations, and enhanced solar irradiance. The starting point of this work was the replication of the fabrication protocol of TiO_2_/FTO photoelectrodes and cell assembly for Zea mays and Bixa orellana.^37,40^ Replicating that protocol with a new sensitizer required identifying and correcting several failure points over successive batches: the electrolyte concentrations were tested in the millimolar range to define a working composition; the platinum counter electrode, initially prepared by pouring the chloroplatinic acid solution, produced a dark, non-uniform layer and was therefore deposited with a home-built spin-coating prototype; and the extraction and sensitization conditions were varied systematically (Table 1). Of the more than 300 cells fabricated during this optimization, many showed a very low response and were discarded, which is why the results below refer to selected devices. No data, characterization results or interpretations from previously reported dyes (Zea mays, Bixa orellana, ayrampo) are reused here.

**Table 1:** Experimental conditions and number of devices for each cell label (C1–C6)

	C1	C2	C3	C4	C5	C6
Use	Lab	Lab	Lab	Lab	Flight	Flight
Material	Powder (P)	Powder (P)	Leaves (L)	Leaves (L)	(P)	(L)
Solvent	EtOH	EtOH	EtOH	EtOH	EtOH	EtOH
Mass (g)	3	2	3	2	3	3
Volume (mL)	60	50	60	50	60	60
Immersion of the best cell (h)	48	48	48	36	48	48
Cells (n)	6	6	6	6	1	1
Batches	3	3	3	3	1	1

The extracts and photoanodes were also characterized by UV-vis, FTIR, XRD, SEM-EDS and IPCE, and two mullaca-sensitized devices (C5 and C6) were evaluated in a stratospheric flight. The new knowledge reported here is therefore the photovoltaic response of mullaca extracts as a function of extraction and immersion conditions, spectroscopic evidence consistent with pigment-TiO_2_ interaction, and preliminary evidence of electrical operation under high-altitude conditions. Theoretical studies by other groups have applied computational design to photoactive molecules for organic solar cells, for example donor–π–acceptor macrocycles^41^ and non-fullerene acceptors,^42^ as well as graphene quantum dots, metal-oxide nanoclusters and polyoxometalate hybrids for nonlinear-optical and sensing applications.^43–45^ In contrast, the present work is purely experimental and explores plant-based natural sensitizers.

Accordingly, this study investigates the optical, structural, and photovoltaic properties of chlorophyll-rich extracts from *Physalis angulata* L. as sensitizers for DSSCs. The optical response of the extracts, their interaction with nanostructured TiO_2_ photoelectrodes, and the photovoltaic performance of the resulting devices were systematically evaluated under simulated solar illumination. Finally, two mullaca-sensitized DSSCs (C5 and C6) were evaluated during a stratospheric flight to assess their electrical response under extreme environmental conditions.

## Materials and methods

### Materials

The main fabrication and assembly steps are summarized in Fig. 1. Fresh leaves of *Physalis angulata* L. (mullaca) were collected in the Loreto region, Peru, and stored in sealed opaque bags at room temperature until processing to minimize pigment photodegradation.

**Fig. 1:**
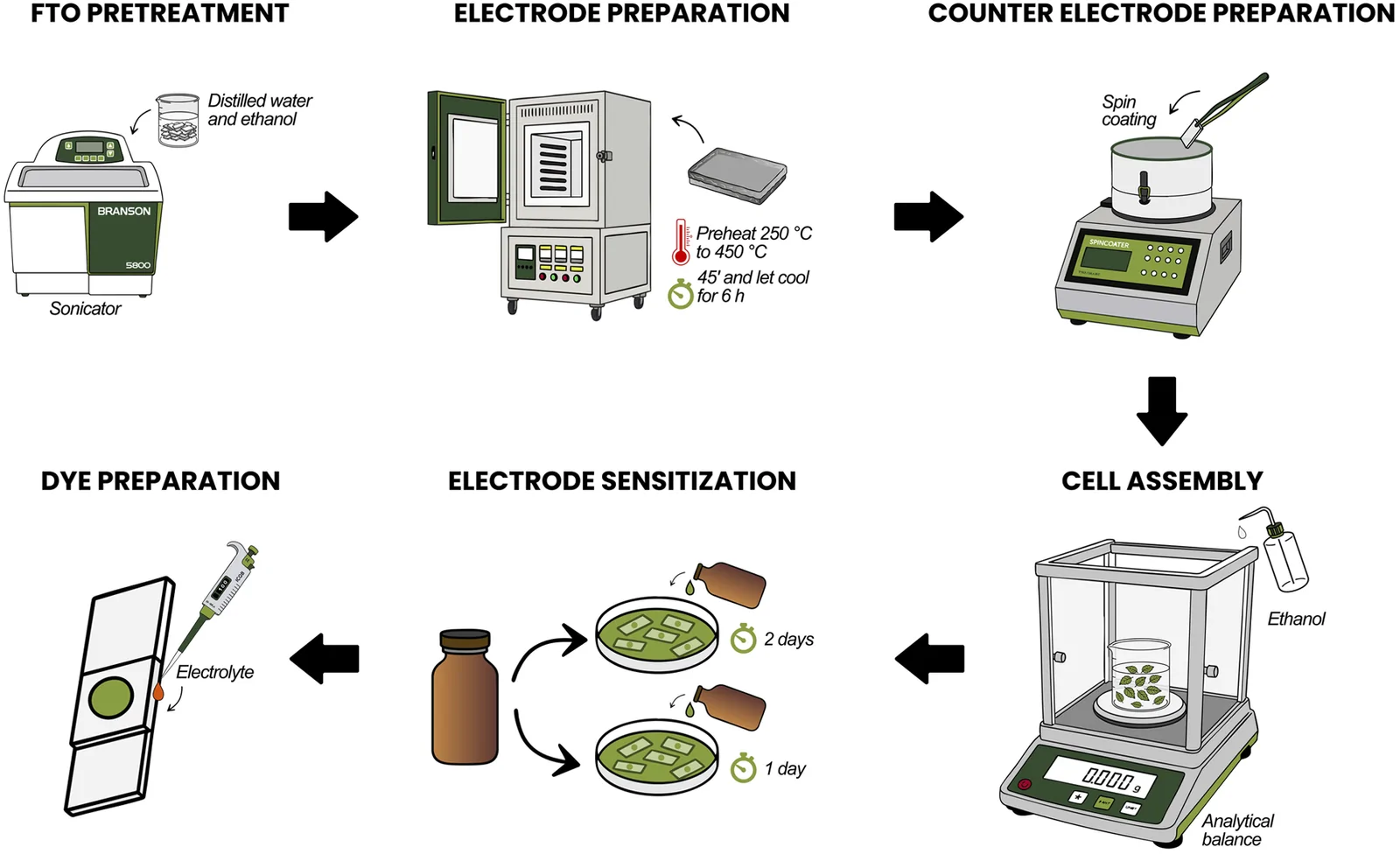
Schematic of the experimental procedure for the fabrication and assembly of the dye-sensitized solar cells.

Fluorine-doped tin oxide (FTO)-coated conductive glass substrates (8–15 Ω sq^−1^) were used as transparent conducting electrodes. Nanostructured TiO_2_ films were prepared by the doctor-blade method from a commercial TiO_2_ paste (Solaronix, Ti-Nanoxide T/SP, code 14412), whereas Pt counter electrodes were fabricated by spin coating a H_2_PtCl_6_·6H_2_O precursor solution in ethanol onto FTO glass (see Preparation of Pt counter electrodes).^46^ Ethanol, methanol, isopropanol, acetonitrile, and distilled water were evaluated as extraction solvents. The electrolyte consisted of an I^−^/I_3_^−^ redox couple dissolved in acetonitrile containing 0.6 M 4-*tert*-butylpyridine, 0.05 M I_2_, and 0.1 M LiI, following standard DSSC formulations reported in the literature.^37,40^

### Preparation of mullaca pigment extracts

Pigments were extracted from two plant materials, leaves and dried mullaca powder, both from the Loreto region (Iquitos), Peru. Fresh leaves were washed, air-dried, and cut into small pieces, whereas the powdered samples were obtained by drying the leaves at approximately 60 °C overnight and grinding them manually in a mortar.

Samples (2 or 3 g) were extracted with 50 or 60 mL of ethanol (96%) by magnetic stirring at 400 rpm for 4–6 h, followed by an overnight rest (total extraction time 12, 24, 36 or 48 h), at room temperature, in the dark, in amber vials wrapped in aluminum foil. The extracts were filtered (Whatman No. 41) and centrifuged to remove suspended particles that could contaminate the TiO_2_ electrode during immersion and were stored at 4 °C between extraction and device fabrication.^37,47^

Combinations of sample mass (2 and 3 g), solvent volume (50 and 60 mL) and extraction time (12–48 h) were investigated to evaluate pigment extraction efficiency and optical properties (Table 1).

Extracts in methanol, isopropanol, acetonitrile and distilled water were prepared following the same procedure (2 g in 50 mL and 3 g in 60 mL, using leaves and powder) and left to rest for approximately 24 h in the dark. Ethanol was selected as the main solvent because the devices sensitized with ethanolic extracts gave the highest power conversion efficiency (0.21%, compared with 0.07% for methanol).

### Preparation of TiO_2_/FTO films

FTO substrates were ultrasonically cleaned in distilled water and ethanol (15 min each). TiO_2_ films were deposited by the doctor-blade technique using the Solaronix paste, over an active area of 0.283 cm^2^ and sintered at 450 °C for 45 min after a preheating step at 250 °C. The electrodes were then allowed to cool gradually to room temperature before sensitization.^47^

### Preparation of Pt counter electrodes

The Pt precursor solution was prepared by dissolving 0.1691 g of H_2_PtCl_6_·6H_2_O in 25.76 g of ethanol (≈32.7 mL; ≈10 mM). A thin film was formed by spin coating, covering the whole conductive area of the FTO substrates. The substrates were rotated at 3000 rpm in a home-built spin-coating prototype, and three drops of the solution were applied onto the conductive side, which was kept spinning for 15 s until the film was uniform.^46^ The electrodes were placed on a ceramic plate and heated in a muffle furnace from 250 to 450 °C, held at 450 °C for 15 min to sinter the platinum layer, and then cooled in a controlled manner to avoid damaging the glass.^46,47^ The counter electrodes were stored protected from air and light and were reactivated before cell assembly.^46^

### Sensitization of TiO_2_

To evaluate the influence of dye adsorption time on photovoltaic performance,^48^ the TiO_2_ photoelectrodes were immersed in the ethanolic mullaca extract for 24, 36 and 48 h in the dark at room temperature. After sensitization, the electrodes were gently rinsed with ethanol to remove loosely adsorbed dye. The optimal extraction and sensitization conditions were selected based on the photovoltaic performance of the resulting DSSCs.^47^

### Assembly of DSSCs

The DSSCs were assembled in a sandwich configuration using a Surlyn spacer (∼60 µm). The I^−^/I_3_^−^ electrolyte was introduced by capillary action, and the cells were thermally sealed.^46^

### Cell nomenclature, experimental conditions, and number of devices

Labels C1–C4 identify the four best laboratory devices, which differ in the plant material (leaves or dried powder) and in the immersion (sensitization) time, as detailed in Table 1; C5 and C6 identify the two mullaca-sensitized devices flown on the stratospheric mission. All devices share the same TiO_2_ film (doctor blade), Pt counter electrode, I^−^/I_3_^−^ electrolyte and active area (0.283 cm^2^). Extracts for all cells were prepared in ethanol (Table 1). Laboratory measurements were performed at 25 °C and 1 atm.

The laboratory values correspond to the best-recorded cell of each condition (the immersion time of the best cell is shown in the table). For C1–C4, six cells per condition were retained (two cells for each of three immersion times, 24, 36 and 48 h; 24 cells in total), and each immersion time corresponds to a separate preparation set. More than 300 cells were fabricated during the optimization of the extraction and sensitization conditions; many showed a low response (*e.g.*, an acceptable *V*_OC_ or FF but a very low *J*_SC_ and efficiency) and were discarded without being recorded. The reported values therefore refer to selected cells, not to all fabricated devices, and the maximum efficiency (0.21%) is a best-case value.

For the flight, nearly 50 cells were prepared; those damaged or degraded during transport from Lima to the launch facility were discarded, and the remaining ones were wrapped in aluminum foil and kept in darkness and under vacuum until integration. One cell of each type (C5 and C6) flew.

### Optoelectronic characterizations

#### UV-vis spectroscopy

UV-vis absorption spectra of the mullaca extracts were recorded in the 300–800 nm range using an Ocean Optics USB4000 spectrophotometer equipped with a UV-vis-NIR light source (DH-2000-BAL) and quartz cuvettes with a 1 cm optical path length. The extracts were prepared in five solvents (methanol, ethanol, isopropanol, water, and acetonitrile) from both dried powder and fresh leaves, using two sample mass-to-solvent volume ratios (2 g/50 mL and 3 g/60 mL), and were left to rest in the dark for approximately 24 h to allow the release of the pigments. Before measurement, the extracts were diluted with distilled water at an extract: water volume ratio of 1 : 3 (v/v). Distilled water was used as the blank.^47^

From the spectra, the characteristic absorption bands of the pigments were identified, and an apparent chlorophyll content was estimated for the alcoholic extracts from the maximum absorbance of the red band (663–666 nm) using the Beer–Lambert law, *c* = *A*/(*ε*l), with solvent-specific absorption coefficients of chlorophyll a taken from Wintermans and De Mots^49^ (ethanol) and Porra *et al.*^50^ (methanol). Because these coefficients correspond to the pure solvents and the extracts were diluted before measurement, the resulting values are indicative and are used only to compare the extracts.

#### Fourier transform infrared spectroscopy (FTIR)

Fourier transform infrared spectroscopy (FTIR) was performed using a Thermo Fisher Nicolet iS10 spectrometer in ATR mode over the range 4000–500 cm^−1^, analyzing TiO_2_ samples, *Physalis angulata* L. powder, and powder scraped from TiO_2_ films after sensitization with mullaca. A total of nine spectra were recorded, corresponding to three replicates for each sample type, to obtain reproducible vibrational responses. The spectra allowed identification of bands associated with functional groups such as O–H, C–H, C=O, and Ti–O, and evaluation of their possible involvement in dye anchoring onto the semiconductor surface, consistent with FTIR studies of natural pigments on TiO_2_.^47^

#### X-ray diffraction (XRD)

X-ray diffraction measurements were carried out on the FTO substrate and on the TiO_2_ films using a Bruker D8 Advance diffractometer equipped with a copper anode (Cu Kα radiation, *λ* = 1.5406 Å). Diffraction patterns were recorded over a 2*θ* range from 10° to 80° with a step size of 0.02° and a counting time adjusted for the identification of crystalline phases. A total of five samples were analyzed: one FTO substrate and four TiO_2_ films. Phase identification was performed by comparison with the JCPDS cards 21-1272 (anatase TiO_2_) and 41-1445 (SnO_2_). For presentation, the patterns were normalized and smoothed in origin.^47^

#### Scanning electron microscopy (SEM) and energy-dispersive X-ray spectroscopy (EDS)

The surface morphology of TiO_2_ films deposited on FTO was analyzed using scanning electron microscopy with a Zeiss EVO 10 instrument, obtaining micrographs at typical magnifications of 50 kx and a scale of 400 nm to evaluate coating homogeneity, the presence of defects, and the porous texture of the surface. A total of four TiO_2_ films on FTO were studied, recording several representative areas for each sample.

On the same samples, energy-dispersive X-ray spectroscopy (EDS) was carried out using the detector integrated into the SEM at an accelerating voltage of 20 kV. Elemental mapping and point analysis were performed on the top surface of the TiO_2_ film deposited on FTO glass, in two analyzed areas (*n* = 2), in order to determine the composition of the films and confirm the presence of titanium and oxygen, as well as the possible incorporation of impurities associated with the substrate or processing residues.^47^

#### Current–voltage (*J*–*V*) curves

Current–voltage (*J*–*V*) curves of the solar cells were obtained using the Toolbox system developed by Dyenamo, with a Uniscan PG580 potentiostat, under simulated solar illumination provided by the LED light source of the system. The LED bias current (400 mA) was set so that the incident intensity, calibrated with a first-generation (crystalline) silicon reference cell, corresponded to a nominal 100 mW cm^−2^ (1000 W m^−2^, 1 Sun) at the sample position. The LED spectrum was not verified against AM 1.5 G, so the efficiencies refer to this nominal illumination. The analysis focused on DSSCs sensitized with mullaca dye, with an active area defined by a circular aperture of 0.283 cm^2^.^47^

Measurements were performed at 25 °C and atmospheric pressure (1 atm). The voltage was swept from 0.65 V to 0 V (reverse direction, from near *V*_OC_ toward short circuit) in steps of 5 mV with a step time of 0.20 s (nominal scan rate 25 mV s^−1^) and no wait time.

From the *J*–*V* curves, the open-circuit voltage (*V*_OC_), short-circuit current density (*J*_SC_), fill factor (FF) and power conversion efficiency (*η*) were determined, with FF = *P*_max_/(*V*_OC_ × *J*_SC_) and *η* = (*V*_OC_ × *J*_SC_ × FF)/*P*_in_ × 100%, where *P*_in_ = 100 mW cm^−2^.^47^

For each condition (C1–C4), the *J*–*V* curves of six cells were measured (*n* = 6 per condition; 24 cells in total). The six cells correspond to two cells for each of the three immersion times (24, 36 and 48 h), and each immersion time corresponds to an independent preparation batch (three batches per condition). Table 6 presents the best-performing cell for each condition, as well as the mean ± standard deviation (SD) of the six recorded cells; the mean therefore includes the effect of the immersion time.

#### Incident photon-to-current efficiency (IPCE)

The incident photon-to-current efficiency (IPCE) spectra of the mullaca-sensitized cells were measured with the Dyenamo IPCE system, consisting of a 175 W xenon lamp, a monochromator, a filter wheel, a set of lenses mounted on an optical rail and a data acquisition system. The system was used with the instrument's own calibration. Spectra were recorded over approximately 350–800 nm.^47^

#### Stratospheric tests

To evaluate the performance of a dye-sensitized solar cell (DSSC) sensitized with mullaca dye under extreme conditions, two mullaca-sensitized cells (C5 and C6; Table 1) were integrated into the Astrotracker payload (Fig. 2a) and flown on the HASP 2024 (High Altitude Student Platform) mission, a joint initiative of NASA's Balloon Program Office and the Louisiana Space Consortium at Louisiana State University. HASP employs a helium-filled, thin-film polyethylene balloon (≈11 million ft^3^) (Fig. 2b) to transport multiple student-built payloads to near-space altitudes.

**Fig. 2:**
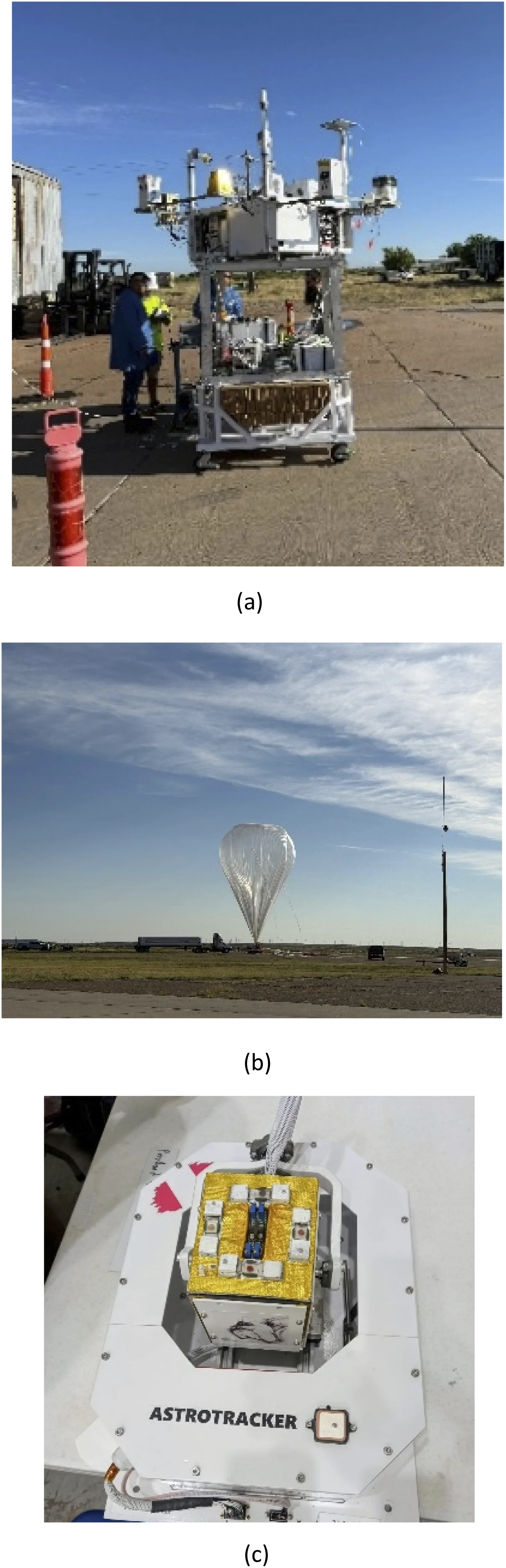
Experimental setup of the mullaca-sensitized DSSC during the stratospheric campaign: (a) integration of the Astrotracker payload on NASA's HASP gondola on the launch site, (b) HASP scientific balloon during ascent, and (c) nanosatellite structure with the Astrotracker module, highlighting the mullaca-sensitized DSSC mounted on its upper face.

The mission was launched from the NASA Columbia Scientific Balloon Facility (CSBF) in Fort Sumner, New Mexico. The platform ascended at approximately 1000 ft min^−1^ to a float altitude of ∼36 km (≈120 000 ft), maintaining flight for approximately 18 h. Laboratory reference conditions were 25 °C and 1 atm (≈1013 hPa). At launch, the pressure was ≈880 hPa and the external temperature (RTD PT100 on the upper face of the payload) ≈21 °C; the pressure fell to ≈60 hPa within the first two hours and remained at a few hPa during float, while the external temperature dropped to ≈ −43 °C at the end of the ascent and reached ≈+56 °C in the afternoon (Table 2). These conditions enabled *in situ* evaluation of DSSC performance under combined low-temperature, low-pressure, and high-altitude exposure.

**Table 2:** Reference and flight conditions (HASP 2024, fort sumner, NM). Flight values are approximate readings from the payload records

Parameter	Lab ref.	At launch	Extreme in flight
Pressure	1 atm (≈1013 hPa)	≈880 hPa	≈60 hPa after 2 h; a few hPa at float
External temp. (PT100, top face)	25 °C	≈21 °C	≈−43 °C/≈+56 °C
OBC board temp. (BME280)	25 °C	≈24 °C	≈+17 °C/≈+56 °C
Relative humidity	—	≈50%	≈2% at end of record
Duration/altitude	—	—	≈18 h; ≈36 km float

Cells C5 and C6 were mounted on the upper face of the nanosatellite (Fig. 2c), under direct natural sunlight and exposed to the external environment. The *I*–*V* curve of each cell was obtained by sweeping the load with a MOSFET stage, with the current measured by an ACS712 current sensor integrated into the onboard computer (OBC) of the Astrotracker payload. During the 18-hour flight, the payload transmitted a report approximately every 15 min (the acquisition was not strictly periodic), each containing around 200 records: the voltage and current produced by the mullaca cells under natural solar irradiation and environmental variables such as temperature, pressure, and relative humidity.

The *I*–*V* curve of each cell used to extract the parameters in Table 7 (≈500 points per cell) was constructed from the records acquired during the three flight periods (ascent, float, and descent), to represent the behavior of the cells over the whole flight rather than at a single instant; it is therefore a composite curve and not a single sweep. The *I*–*V* data were smoothed in origin using a Savitzky–Golay filter (10-point window, polynomial order 2) prior to parameter extraction; *V*_OC_, *I*_SC_ and *P*_max_ were taken from the smoothed curves.

The unsmoothed data and the time series of voltage, current, temperature and power recorded during the flight are provided in the data repository (see Data availability).

## Results and discussion

### Optical roperties by UV-visible spectroscopy

UV-vis spectroscopy was used to evaluate the optical properties of the *Physalis angulata* L. (mullaca) extracts and their suitability as natural sensitizers for DSSCs. The spectra exhibited well-defined absorption bands in the visible region, with principal maxima near 538, 610 and 666 nm (Fig. 3), consistent with chlorophyll-containing extracts and with values reported in the literature; without chromatographic separation, these bands do not establish the identity or relative abundance of individual pigments.

**Fig. 3:**
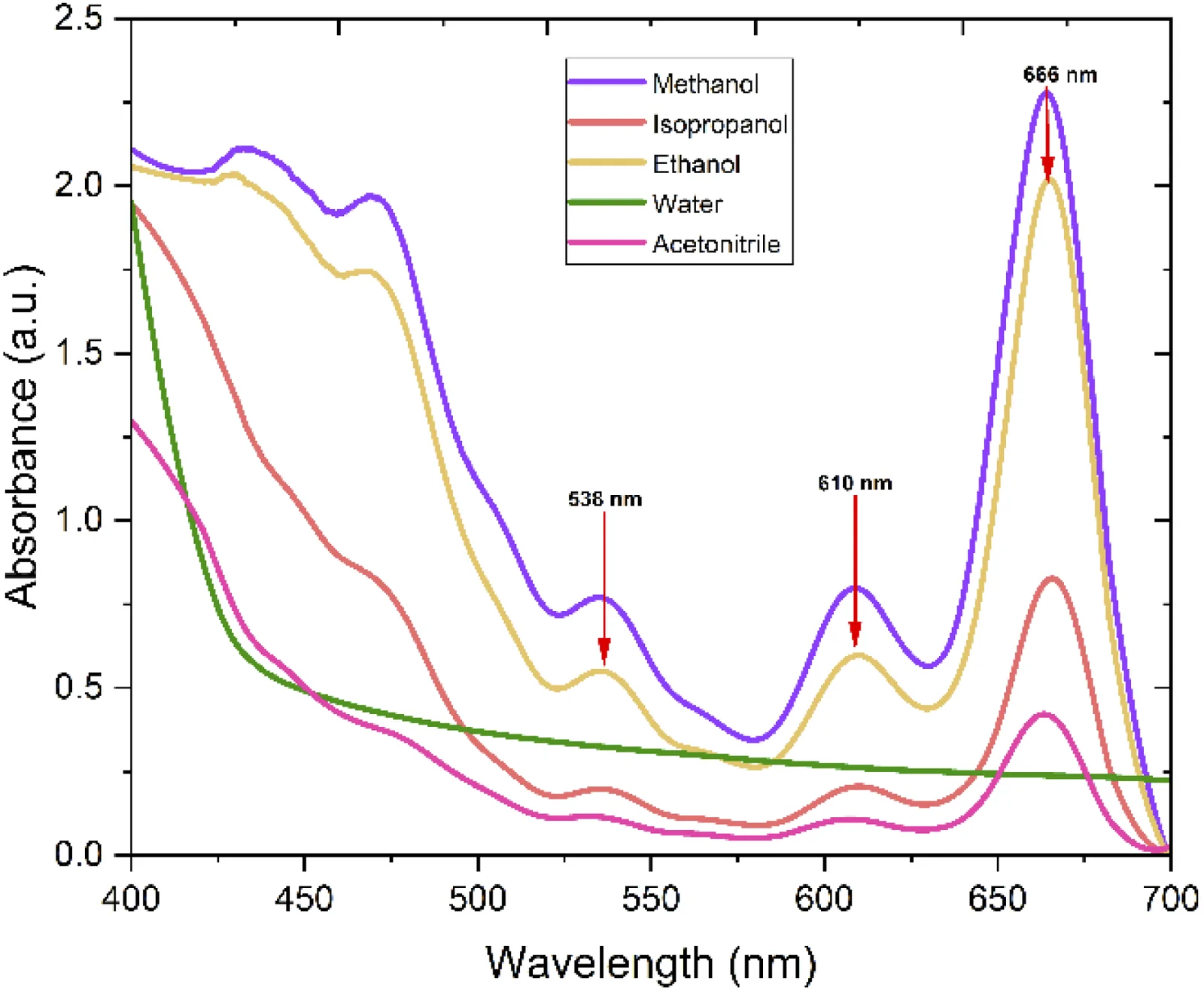
UV-vis absorption spectra of *Physalis angulata* L. extracts in methanol, isopropanol, ethanol, water and acetonitrile, recorded between 300 and 800 nm and plotted between 400 and 700 nm. Extracts were diluted with distilled water at an extract: water volume ratio of 1 : 3 (v/v) and measured against distilled water as the blank. The principal absorption maxima of the alcoholic extracts appear near 538, 610 and 666 nm; arrows mark those of the ethanolic extract.

From the maximum absorbance of the red band (663–666 nm), an apparent chlorophyll content was estimated for the alcoholic extracts using the Beer–Lambert law, *c* = *A*/(*ε*l), with a 1 cm path length and solvent-specific absorption coefficients of chlorophyll *a* of 83.4 L g^−1^ cm^−1^ at 665 nm in 96% ethanol^49^ and 80.0 L g^−1^ cm^−1^ at 665 nm in methanol,^50^ yielding 24.6 mg L^−1^ for ethanol and 27.3 mg L^−1^ for methanol (Table 3). No coefficients are available for isopropanol, acetonitrile, or water, for which only the absorbance is compared. These values are only indicative because they assume that the red band is due to chlorophyll *a* alone and neglect other absorbing species and scattering; they should therefore be regarded as comparative indicators rather than absolute concentrations.

**Table 3:** Optical properties and apparent chlorophyll content (comparative indicator) of *Physalis angulata* L. extracts in different solvents. Measurements were made on extracts of ground leaves (3 g in 60 mL of solvent), diluted 1 : 3 (v/v) with distilled water before recording; *A*_max_ is the maximum absorbance read from the spectrum with the Ocean View software. The apparent chlorophyll concentration (c) was calculated with the Beer–Lambert law, *c* = *A*/(*ε*·l), only for methanol and ethanol, for which specific absorption coefficients are available (80.0 and 83.4 L g^−1^ cm^−1^, respectively); for isopropanol, acetonitrile, and water no coefficients are available, so only the absorbance is compared (n.d. = not determined)

Solvent	Absorption peaks (nm)	*A*_max_ (a.u.)	*c* (mg L^−1^)
Methanol	573.32	2.18	27.3
	608.03		
	663.39		
Ethanol	538.32	2.05	24.6
	610.35		
	666.04		
Isopropanol	539.51	0.93	n.d.
	608.80		
	664.90		
Acetonitrile	606.67	0.45	n.d.
	663.96		
Water	—	0.24	n.d.

Alcoholic solvents yielded the highest absorbance and apparent chlorophyll concentrations, whereas isopropanol, acetonitrile and water produced weak signals close to the baseline and markedly lower pigment contents. Methanol and ethanol were therefore compared as extraction solvents in complementary device tests.

Although the methanolic extract presented a higher absorbance, its devices gave a lower fill factor and efficiency than those prepared with the ethanolic extract (*η* = 0.07% for methanol *vs.* 0.21% for ethanol). This contrast indicates that a higher absorbance in solution does not directly translate into a higher conversion efficiency, as additional factors are involved, such as pigment stability, extract composition, and the efficiency of dye anchoring onto the TiO_2_ surface during sensitization. Consequently, ethanol was selected as the extraction solvent for the devices reported in Table 6, as it offered a more favorable balance between pigment extraction and its effective use in current generation. Thus, the UV-vis response, together with the *J*–*V* results, indicates that mullaca provides an absorption spectrum complementary to that of TiO_2_, extending photon harvesting into the visible region and contributing to photocurrent generation under simulated solar illumination.

### Chemical interactions by FTIR

Following the optical characterization, FTIR spectroscopy was employed to investigate the chemical interactions between the mullaca pigments and the TiO_2_ photoelectrode. Understanding these interactions is essential because efficient dye adsorption directly influences electron injection and the overall photovoltaic performance of DSSCs. The TiO_2_ spectrum showed an intense peak at 3539.13 cm^−1^, attributed to the stretching of surface hydroxyl (O–H) groups, which can act as active sites for interaction with the dye. Additionally, bands between 2938.64 cm^−1^ and 2077.45 cm^−1^ were observed, associated with aliphatic C–H vibrational modes and possible combinations of stretching and overtone bands. In the low-frequency region, bands between 826 cm^−1^ and 753 cm^−1^ correspond to Ti–O vibrational modes characteristic of the anatase phase, consistent with reports for nanostructured TiO_2_ electrodes.^8,51^

The spectrum of the *Physalis angulata* L. powder (Fig. 4b) revealed a set of bands associated with functional groups typical of a multipigment organic matrix: a dominant contribution in the 3300–3400 cm^−1^ region attributed to O–H/N–H stretching, aliphatic C–H signals around 2919 and 2854 cm^−1^, and a strong band at 1729.9 cm^−1^ corresponding to carbonyl (C=O) groups. Additionally, bands near 1614 cm^−1^ were identified, associated with aromatic or conjugated C=C bonds, and multiple signals in the 1373–1024 cm^−1^ range assigned to C–O and C–N vibrations. This indicates the presence of chlorophylls and phenolic compounds with polar functional groups potentially capable of interacting with the oxide surface.^51–54^

**Fig. 4:**
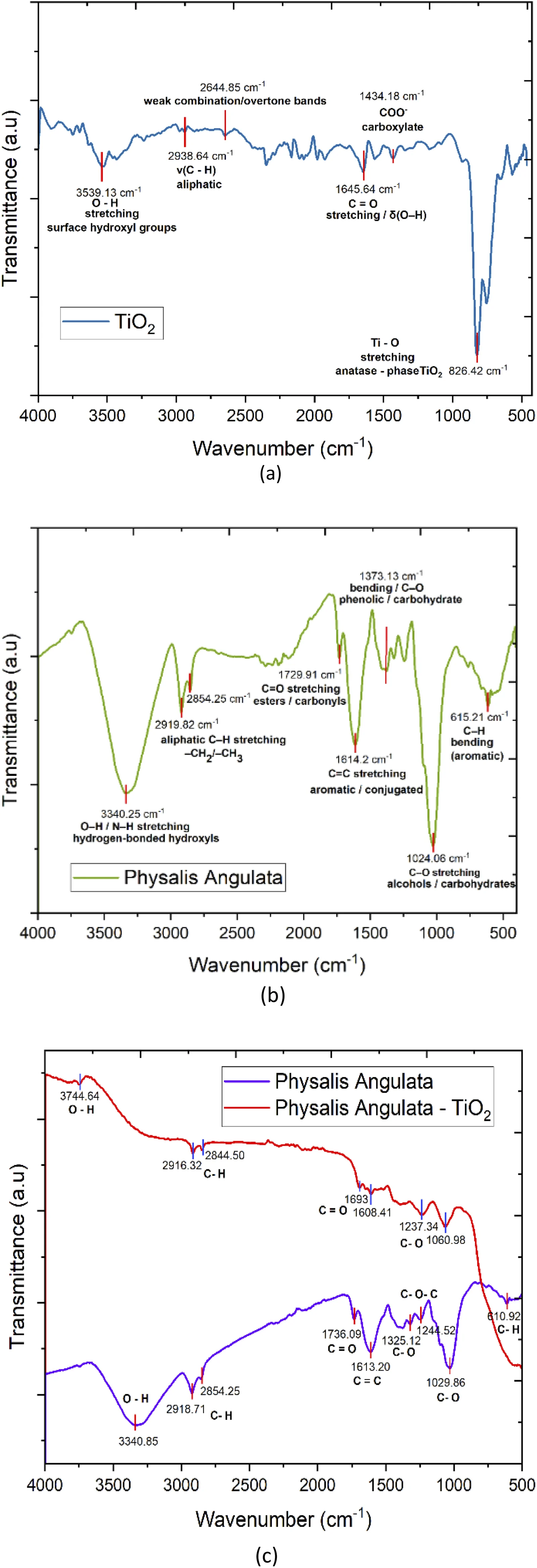
FTIR spectra (ATR, 4000–500 cm^−1^) of (a) the TiO_2_ film deposited on FTO, (b) *Physalis angulata* L. powder, and (c) powder scraped from TiO_2_ films after sensitization with mullaca. Selected band positions (cm^−1^) are indicated; the replicate variation in band position is ≈6 cm^−1^.

After sensitization, shifts and intensity changes were observed in several FTIR bands, particularly in the carbonyl and C–O regions (Fig. 4c), where the *Physalis angulata* powder is compared with the powder scraped from the TiO_2_ film sensitized with *Physalis angulata*, both measured in the same run. In the powder, the carbonyl band at 1736 cm^−1^ shifted to 1693 cm^−1^ in the sensitized film powder (−43 cm^−1^), and the C–O band at 1030 cm^−1^ moved to 1061 cm^−1^ (+31 cm^−1^), while the C–O–C band at 1245 cm^−1^ shifted by only −7 cm^−1^ (1237 cm^−1^). The C–H (2919 → 2916 and 2854 → 2845 cm^−1^) and C=C (1613 → 1608 cm^−1^) bands shifted by only a few cm^−1^. The same band appears at slightly different positions in replicate measurements of the powder (*e.g.*, C=O at 1729.9 cm^−1^ in Fig. 4b and 1736.1 cm^−1^ in Fig. 4c, a difference of ≈6 cm^−1^), so only shifts clearly larger than this variation are considered meaningful.

The O–H band of the sensitized film appeared at 3745 cm^−1^, whereas the pigment O–H band appeared at 3341 cm^−1^; these correspond to different species (surface Ti–OH and organic O–H, respectively) and are therefore not compared as a shift. These spectral modifications suggest interactions between oxygen-containing functional groups of the mullaca pigments (C=O and C–O) and surface Ti–OH groups, which may be consistent with carboxylate- or ester-type bonding, in agreement with what has been reported for other natural and synthetic dyes adsorbed on anatase.^52,53^

Overall, the band assignments indicate that carboxyl and hydroxyl groups of the pigment are plausible candidates for anchoring onto TiO_2_, while aromatic and conjugated functionalities contribute to visible light absorption. These observations are consistent with, but do not prove, a chemical interaction between the pigments and the TiO_2_ surface, because overlapping bands, residual solvent and sample loading may also contribute to the observed shifts.

### X-ray diffraction

X-ray diffraction (XRD) was employed to characterize the crystalline structure of the FTO substrate and the TiO_2_ photoelectrode prior to sensitization. Identifying the crystalline phases is essential because the photovoltaic performance of DSSCs strongly depends on the crystallinity and phase composition of the semiconductor.

The diffraction pattern of the TiO_2_ film deposited on FTO (Fig. 5) showed the characteristic reflections of tetragonal SnO_2_ (rutile-type; JCPDS 41-1445) arising from the fluorine-doped tin oxide substrate, at ≈26.6°, 33.8°, 51.6° and 65.5°, assigned to the (110), (101), (211) and (301) planes. The sharp SnO_2_ reflections indicate a well-crystallized conductive layer, consistent with previous reports on commercial FTO-coated glass used in DSSCs.^55,56^ In addition, the pattern shows reflections at ≈25.3°, 37.8°, 48.0°, 53.9°, 55.1° and 62.7°, which are indexed to the (101), (004), (200), (105), (211) and (204) planes of anatase TiO_2_ (JCPDS card 21-1272).^38,47^

**Fig. 5:**
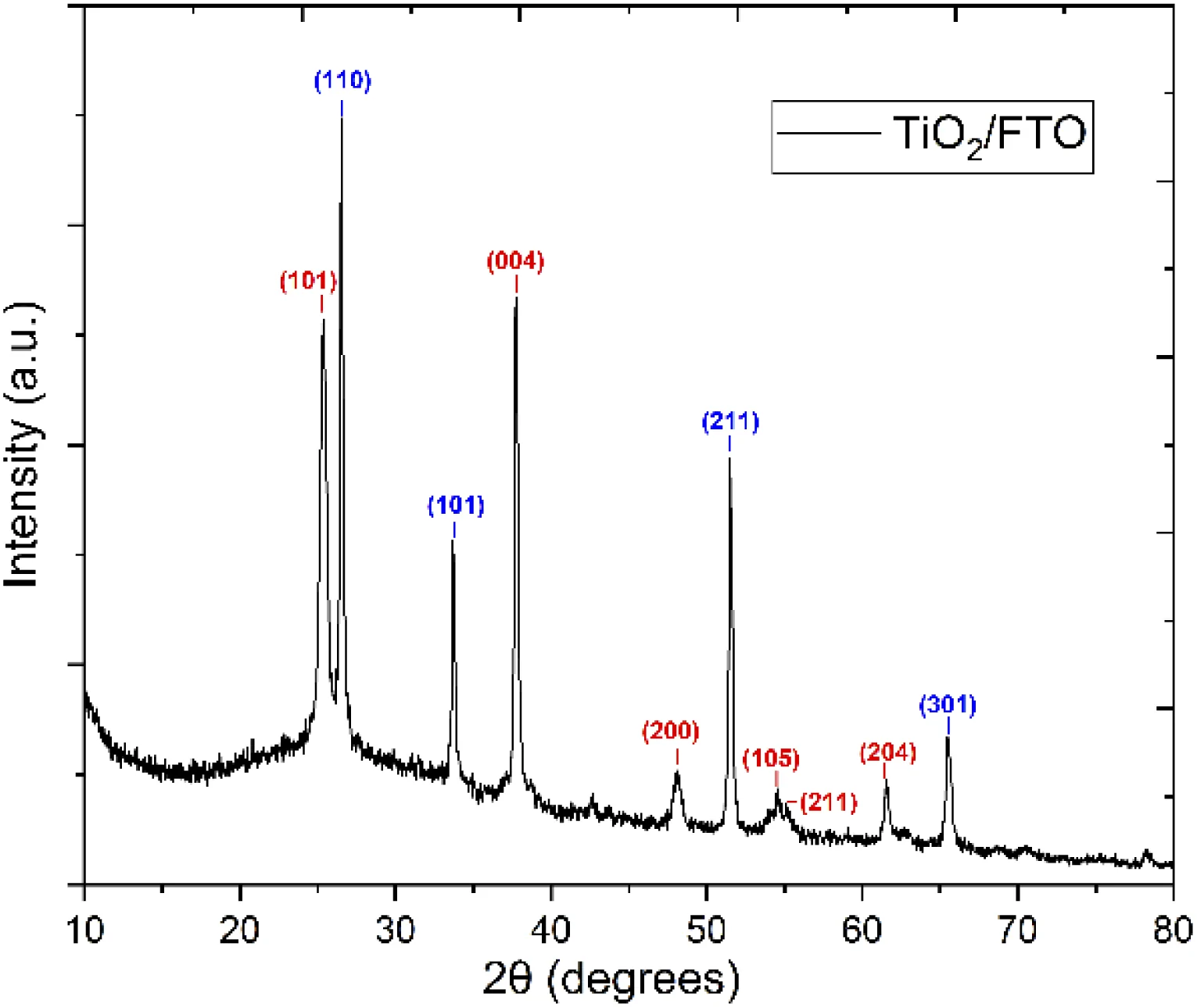
XRD diffraction pattern of the TiO_2_ film deposited on FTO substrate. Blue labels mark the reflections of tetragonal SnO_2_ from the FTO substrate ((110), (101), (211) and (301)); red labels mark the reflections of anatase TiO_2_ ((101), (004), (200), (105), (211) and (204)).

The coexistence of the substrate and anatase reflections is consistent with an anatase TiO_2_ layer deposited on FTO, with the substrate peaks remaining at the same positions. The anatase phase is generally associated with favorable electron transport and dye adsorption in DSSCs;^8^ however, XRD only provides phase information and does not establish surface area, connectivity, defect density or charge-transport efficiency.

### SEM morphology

The morphology and elemental composition of the TiO_2_ photoelectrode were investigated by scanning electron microscopy (SEM) coupled with energy-dispersive X-ray spectroscopy (EDS). These analyses provide complementary information regarding the surface structure, particle distribution, film thickness, and elemental composition of the photoelectrode. Cross-sectional SEM images revealed a continuous TiO_2_ layer with an average thickness of 7.36 ± 0.18 µm (mean ± SD, *n* = 76 measurements from 2 images). Individual thickness values ranged from ≈7.0 to ≈8.0 µm, most of them between 7.1 and 7.6 µm. Surface micrographs showed densely packed nanoparticles with an average diameter of 41.07 ± 7.10 nm (mean ± standard deviation, *n* = 263 particles from 3 images), with sizes ranging from ≈20 to ≈60 nm and a maximum of the size distribution at 40–44 nm, forming a porous nanostructured film.

Only images with adequate resolution were used for the measurements; blurred images were discarded. The micrographs shown in Fig. 6 are representative images. The cross-sectional micrograph confirms a homogeneous layer well adhered to the substrate, without delamination or macroscopic voids, while the top-view image reveals a surface fully covered by densely packed nanoparticles, forming a porous network.

**Fig. 6:**
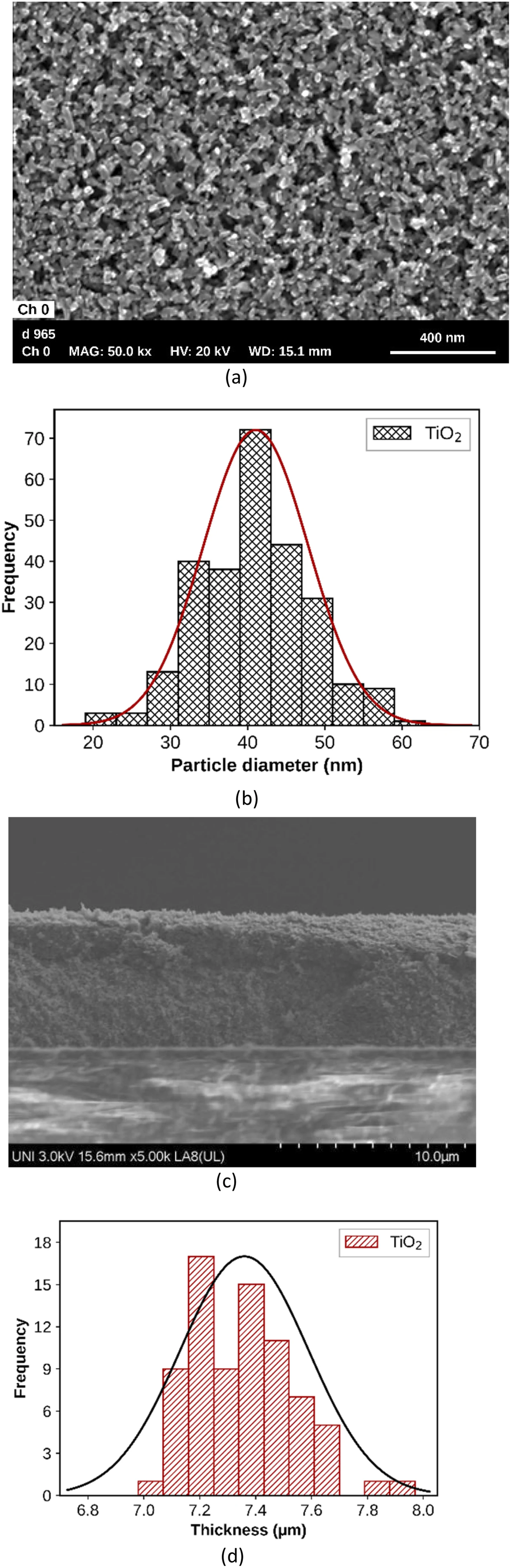
SEM images and size/thickness distributions of the TiO_2_ photoanode: (a) top-view SEM image, (b) particle size distribution, (c) cross-section SEM image and (d) film thickness distribution.

Such a porous nanostructured morphology is generally considered favorable for DSSCs, as it can provide a large surface area for dye adsorption while maintaining interconnected pathways for electron transport.^12^ The observed morphology is therefore compatible with efficient sensitization and charge collection, although the surface area and porosity were not measured in this work.

### Compositional analysis by EDS of the TiO_2_/FTO photoelectrode

EDS elemental mapping (Fig. 7) showed a distribution consistent with a homogeneous distribution of Ti and O throughout the photoelectrode, while Sn signals originated from the underlying FTO substrate. No evidence of localized impurities or elemental segregation was observed within the analyzed area. The contributions of Ti and O are attributed to the nanocrystalline titanium dioxide film, while the Sn signal is associated with the conductive FTO layer of the substrate, in agreement with reports on TiO_2_ coatings deposited on fluorine-doped tin oxide glass, where EDS is used to confirm the presence of both the semiconductor and the conductive substrate without undesired secondary phases.^57,58^

**Fig. 7:**
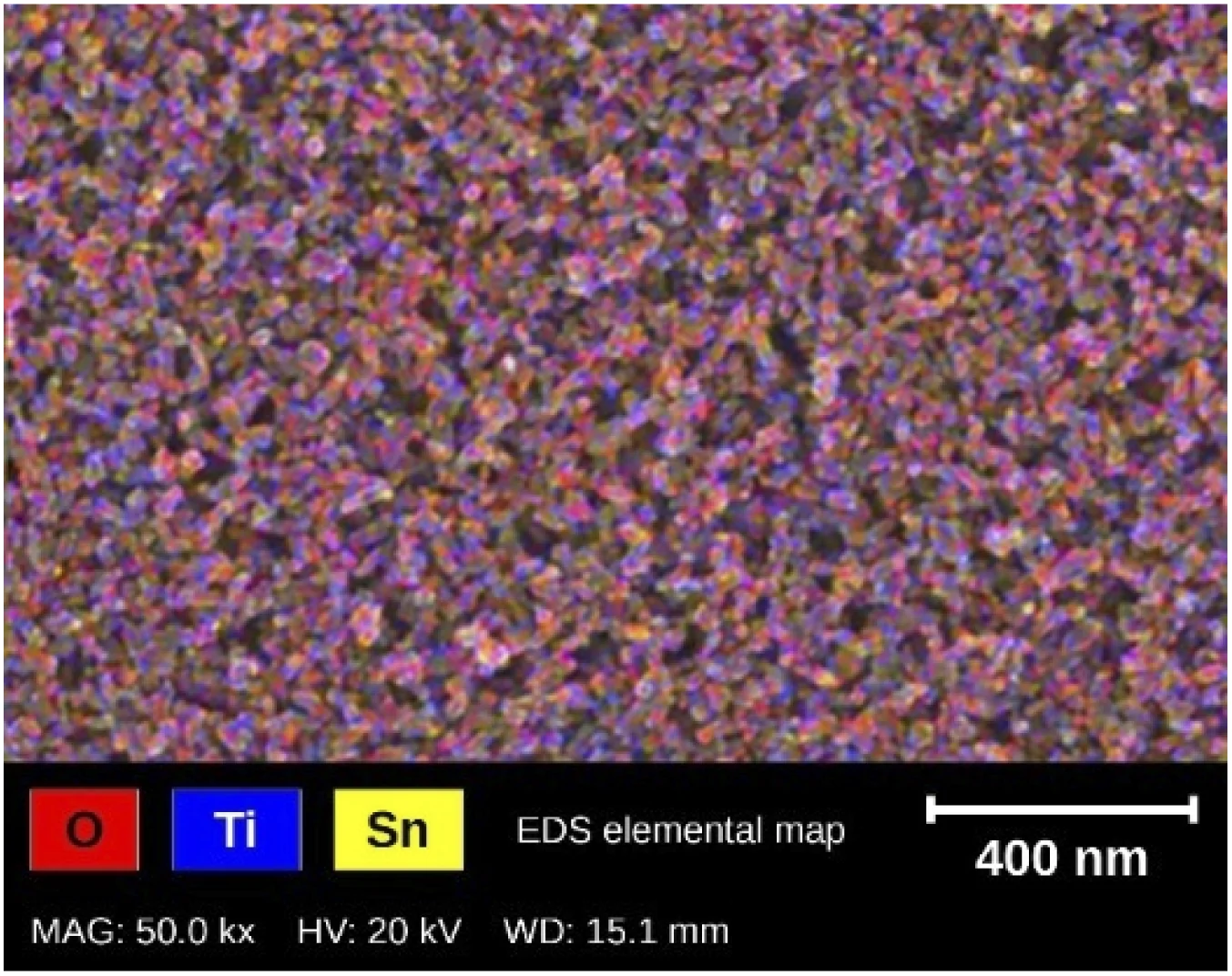
Elemental EDS mapping of the TiO_2_/FTO photoanode surface.

The uniformity of the elemental mapping indicates that the coating is chemically continuous and that no aggregates rich in metallic impurities or TiO_2_-deficient regions were formed within the analyzed areas. These observations are consistent with the SEM images, indicating a chemically uniform TiO_2_ coating over the conductive substrate.

Table 4 presents the elemental composition, expressed in weight percentage (wt%), obtained from the top surface of the TiO_2_ film deposited on FTO glass (accelerating voltage 20 kV; *n* = 2 analyzed areas). The *σ* values correspond to the standard deviation (uncertainty) of each element as reported by the EDS acquisition software, not to the dispersion between the two areas. The analysis is used to characterize the chemical composition of the coating and to verify the contribution of the FTO substrate. Because the interaction volume includes the substrate, these values are semi-quantitative and do not give the exact stoichiometry of TiO_2_, and the absence of other detected elements does not imply total purity.^47^ Overall, the combined SEM and EDS analyses indicate that the TiO_2_ photoelectrode has structural and compositional characteristics consistent with a suitable platform for the fabrication of mullaca-sensitized DSSCs.

**Table 4:** Elemental composition (wt%) of the TiO_2_/FTO photoanode obtained from EDS analysis of the film surface (20 kV; mean of *n* = 2 areas). *σ* = uncertainty reported by the EDS software, not the dispersion between the two areas. Values are semi-quantitative; the FTO substrate contributes to the Sn signal

Element	wt%	*σ* (wt%)
Ti	55.18	0.41
O	39.22	0.44
Sn	5.59	0.12
Total	100	

### Incident photon-to-current efficiency (IPCE)

The spectral distribution of the IPCE is consistent with the optical absorption of the *Physalis angulata*/TiO_2_ system. Ten cells were evaluated; Fig. 8 shows the spectrum of the best-represented device, C3. Its IPCE reached its highest value, ≈25%, at the lower limit of the measured range (≈350 nm), where TiO_2_ itself also absorbs, and decreased to a minimum of ≈7% near 490 nm. A secondary maximum of ≈14% appeared at ≈540 nm, which can be associated with the visible absorption band of the *Physalis angulata* pigments (538 nm in the ethanolic extract; Fig. 3). This coincidence between the IPCE maximum and the visible absorption band of the dye is consistent with Physalis-derived pigments contributing to photocurrent generation in the green region of the spectrum.^40,47^

**Fig. 8:**
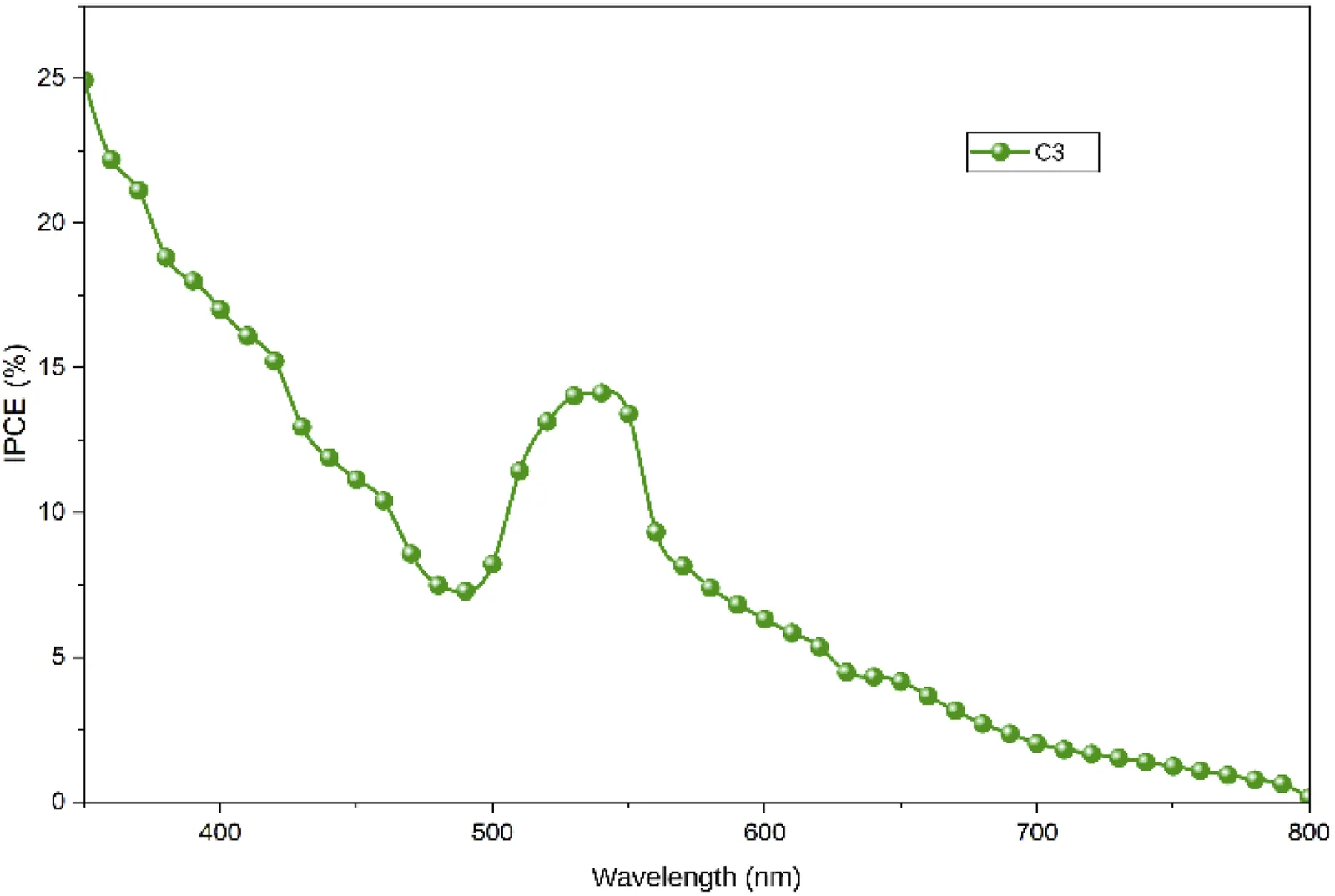
Incident photon-to-current efficiency (IPCE) spectrum of the DSSC sensitized with the leaf extract of *Physalis angulata* L. (cell C3: leaves, 3 g/60 mL, 48 h immersion).

At longer wavelengths, the IPCE decreased monotonically, from ≈6% at 600 nm to <1% at 800 nm, and no distinct feature was observed at the extract absorption bands near 610 and 666 nm. The agreement between the UV-vis and IPCE spectra in the green region, where the secondary IPCE maximum (≈540 nm) coincides with the absorption band of the mullaca pigments, is consistent with the extracted pigments extending the photoresponse of TiO_2_ into the visible region, whereas the response in the red region is weak.

Overall, the combined UV-vis, FTIR, and IPCE results are consistent with a mullaca extract that absorbs visible light and contributes photocurrent in TiO_2_-based cells, supporting its exploration as a natural sensitizer for DSSCs. The photon-to-current conversion is modest (IPCE ≤ 25% over the measured range and ≈14% at the 540 nm band), in line with the low overall power conversion efficiency of *Physalis angulata*-based cells.^22,35,39,40,53^

### Photovoltaic parameters (*J*–*V* curves)

The photovoltaic performance of the mullaca-sensitized DSSCs was evaluated under simulated solar illumination through current density–voltage (*J*–*V*) measurements (Fig. 9a). The photovoltaic parameters obtained under laboratory conditions are summarized in Table 6; the conditions represented by C1–C4 are defined in Table 1.

**Fig. 9:**
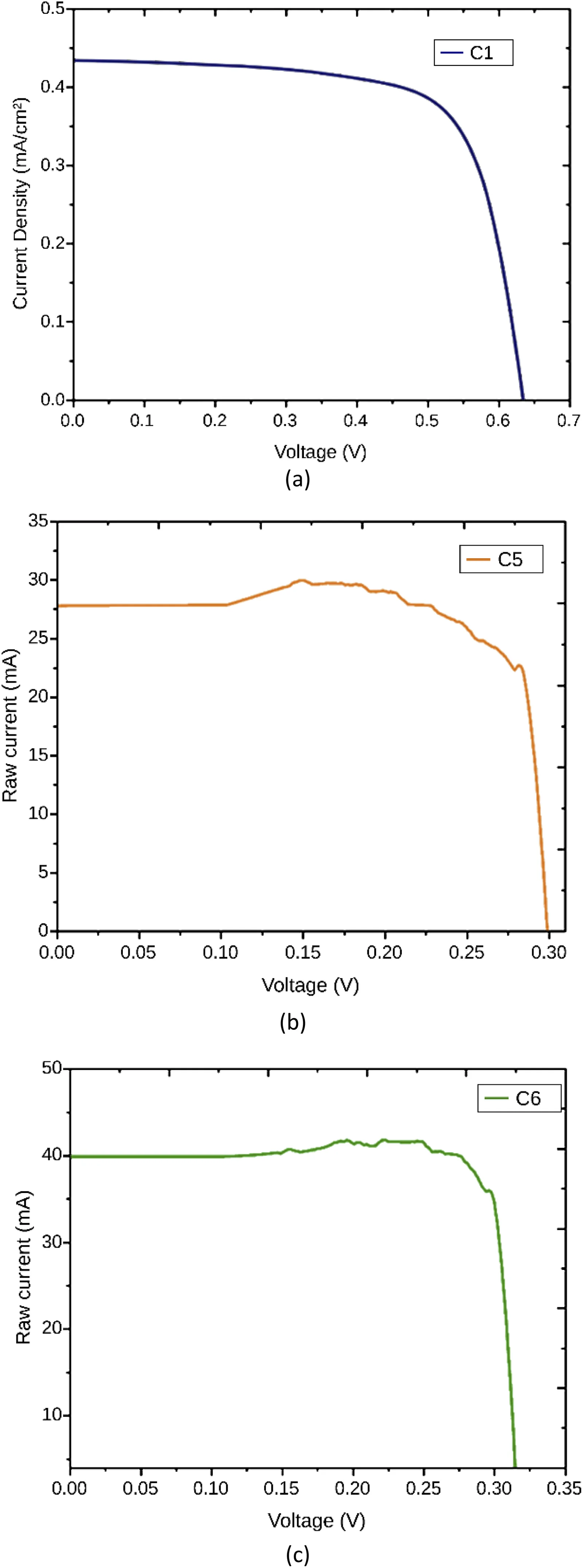
Current density–voltage curve of cell C1 (a) measured in the laboratory under simulated solar illumination, and raw current–voltage curves of cells C5 (b) and C6 (c) measured in flight under stratospheric conditions. In (b) and (c) the current axis shows raw, uncalibrated channel readings.

Table 5 summarizes representative photovoltaic parameters reported in the literature for DSSCs sensitized with selected natural dyes. Although direct comparisons should be interpreted with caution because of differences in device architecture and testing conditions, these data provide a useful benchmark for evaluating the performance of the mullaca-based DSSCs reported below (Table 6).^5,22^

**Table 5:** Photovoltaic parameters of dye-sensitized solar cells using natural photosensitizers^a^

Photosensitizer	*V*_OC_ (V)	FF	*η*%	Reference
Red onion	0.44	0.48	0.14	55
Cherries	0.30	0.38	0.18	56
Lily	0.49	0.66	0.17	57
Dragon fruit	0.54	0.62	0.32	58
Zea mays	0.58	0.59	0.30	39
Bixa orellana	0.60	0.61	0.35	39

a Under controlled conditions, cell C3 (leaves, 48 h immersion) achieved *V*_OC_ ≈ 0.64 V, *J*_SC_ ≈ 0.518 mA cm^−2^, FF ≈ 0.64, and a maximum power conversion efficiency of *η* ≈ 0.21%, whereas cell C1 (powder, 48 h immersion) exhibited *V*_OC_ ≈ 0.635 V, *J*_SC_ ≈ 0.433 mA cm^−2^, FF ≈ 0.71, and *η* ≈ 0.20%.

**Table 6:** Photovoltaic parameters of the laboratory DSSCs (C1–C4; conditions in Table 1) under laboratory illumination (25 °C, 1 atm): best-recorded cell (‘best’) and mean ± SD of the six recorded cells (‘mean’, *n* = 6) of each condition (two cells for each immersion time of 24, 36 and 48 h; the mean therefore includes the effect of immersion time)

Cell	Material	Immersion (h)	*V*_OC_ (V)	*J*_SC_ (mA cm^−2^)	FF	*η* (%)
C1 best	Powder	48	0.635	0.433	0.713	0.20
C1 mean	Powder	24–48 h	0.613 ± 0.027	0.26 ± 0.11	0.68 ± 0.07	0.11 ± 0.05
C2 best	Powder	48	0.610	0.379	0.677	0.16
C2 mean	Powder	24–48 h	0.607 ± 0.027	0.23 ± 0.10	0.68 ± 0.05	0.10 ± 0.04
C3 best	Leaves	48	0.640	0.518	0.643	0.21
C3 mean	Leaves	24–48 h	0.583 ± 0.063	0.24 ± 0.17	0.64 ± 0.12	0.10 ± 0.07
C4 best	Leaves	36	0.635	0.386	0.678	0.17
C4 mean	Leaves	24–48 h	0.590 ± 0.024	0.28 ± 0.11	0.64 ± 0.05	0.10 ± 0.04

The efficiency obtained for the best-performing cell, C3 (*η* ≈ 0.21%, *J*_SC_ = 0.518 mA cm^−2^) is modest, but it lies within the range reported in Table 5 for DSSCs sensitized with natural dyes. Interestingly, the highest photovoltaic performance was obtained with ethanolic extracts rather than with the methanolic extracts, which showed the highest apparent chlorophyll content (Table 3). This suggests that efficient DSSCs require not only effective pigment extraction but also favorable dye adsorption and interfacial charge transfer, although these processes were not directly measured here.^47^

From a practical standpoint, these devices use a low-cost, locally available sensitizer extracted with ethanol, without chemical purification or rare metals, which is relevant for low-cost photovoltaics with a low environmental impact and for educational or remote-site applications.

To explore the electrical operation of the mullaca-sensitized DSSCs under extreme environmental conditions, two devices (C5 and C6) were integrated into the Astrotracker payload and tested during the NASA HASP 2024 stratospheric flight.

During the stratospheric flight, the mullaca-sensitized cells showed a reduced *V*_OC_ of 0.299 and 0.315 V (C5 and C6, respectively; Table 7) compared with 0.61–0.64 V in the laboratory (C1–C4). No efficiency is reported for the flight devices because the incident irradiance was not recorded; this decrease is consistent with, but cannot be uniquely attributed to, the variable irradiance and the extreme pressure and temperature conditions at high altitude.

**Table 7:** Electrical parameters of the two mullaca-sensitized cells (C5 and C6, two different devices; conditions in Table 1) extracted from the smoothed in-flight *I*–*V* curves of a single flight

Cell	*I*_SC_ (mA)^a^	*V*_OC_ (V)	*P*_max_ (mW)	FF	FF^a^
C5	25.9	0.299	6.95	0.90	0.74
C6	38.2	0.315	11.68	0.97	0.82

a Raw readings of the Astrotracker current channel, which was not calibrated against a reference; they are given for completeness only, and absolute currents, current densities and powers are not interpreted. *V*_OC_ was measured directly. Parameters were extracted from *I*–*V* curves of ≈500 points smoothed in origin (Savitzky–Golay filter, 10-point window, polynomial order 2). FF = *P*_max_/(*I*_SC_ × *V*_OC_); FF* = *P*_max_/(*I*_max_ × *V*_OC_), where *P*_max_ = max(*V* × *I*) and *I*_max_ is the maximum measured current of the sweep. Both ratios are independent of a linear current-scale factor. Because the current channel was not calibrated, no power conversion efficiency is reported. Efficiencies estimated from the raw currents are not physically meaningful and are therefore not reported.

Despite the low-pressure environment and large temperature variations experienced during the flight, the devices produced a measurable electrical response during the flight (Fig. 9b and c). Apparent fill factors of 0.90 and 0.97 were obtained for C5 and C6 from the in-flight sweeps; because the measured current increased slightly with voltage, a conservative fill factor, FF* = 0.74 and 0.82, is also reported (Table 7). These values should not be directly compared with laboratory measurements because the electrical operating conditions and natural illumination during the stratospheric experiment differed substantially from the natural sunlight testing conditions.^37^

Although the open-circuit voltage decreased from 0.61–0.64 V in the laboratory to 0.30–0.32 V in flight, the mullaca-sensitized DSSCs continued to generate a measurable voltage and current signal throughout the flight, showing that they remained electrically operational under high-altitude conditions during this single flight. However, because pressure, temperature, and irradiance changed simultaneously, their individual effects cannot be separated without controlled tests, and no post-flight characterization is available; therefore, neither long-term stability, degradation, nor robustness can be concluded from these results.

Relationship between flight and laboratory cells. C5 was prepared like C1 (powder, 3 g, 60 mL, 48 h) and C6 like C3 (leaves, 3 g, 60 mL, 48 h). Their open-circuit voltage in flight (0.299 and 0.315 V) was about half that of C1–C4 at 25 °C and 1 atm (0.610–0.640 V). The flight currents are reported as raw channel readings and are not compared with the laboratory values because the current-sensing chain has not been calibrated; the flight is therefore presented as preliminary evidence of electrical operation and not as a performance comparison.

Limitations: the results come from a single flight of ≈18 h with two mullaca devices. The sensitizer is chemically undefined (no HPLC), *η* = 0.21% does not by itself make it a competitive sensitizer, irradiance and cell temperature were not recorded in flight, the current-sensing chain has not been calibrated against a reference, and no post-flight characterization is available. The payload attitude control did not achieve Sun pointing during the flight, owing to an actuator failure (HASP 2024 final report); the incidence angle of sunlight on the cells is therefore unknown. HASP reproduces low pressure, strong temperature variations and a near-extra-terrestrial solar spectrum, but not particle radiation or orbital thermal cycling. Future work should address reproducibility, efficiency, chemical stability, calibrated *I*–*V* measurements with a reference irradiance, and *I*–*V* and impedance characterization before and after flight. Because each immersion time corresponds to a separate preparation set, the effect of immersion time cannot be separated from set-to-set variation, and the reproducibility between preparations was not evaluated independently.

Looking ahead, the use of plant-derived pigments in photovoltaic devices may benefit from advances in related fields, including spectral reflectance analysis for the rapid, non-destructive quantification of material properties,^59^ low-cost, waste-derived materials and dye-adsorption strategies relevant to pigment loading and recovery^60,61^ and two-dimensional heterojunctions and micro-LED technologies for future optoelectronic integration.^62,63^ Integration of DSSCs with energy-storage devices, such as supercapacitors, is another possible direction.^64^

## Conclusions

Chlorophyll-rich extracts from *Physalis angulata* L. (mullaca) were employed as natural sensitizers for TiO_2_-based dye-sensitized solar cells, achieving a maximum power conversion efficiency of 0.21% under simulated solar illumination (best device, C3: *V*_OC_ = 0.640 V, *J*_SC_ = 0.518 mA cm^−2^, FF = 0.643). The combined UV-vis, FTIR, IPCE, XRD, SEM-EDS, and photovoltaic characterization showed that the extracted pigments absorb in the visible region, show spectral features consistent with interaction with the TiO_2_ surface, and contribute to photocurrent generation. Two devices (C5 and C6) remained electrically operational during a single stratospheric flight (*V*_OC_ = 0.299 and 0.315 V). These results correspond to the best case among the selected cells, and because the flight was a single test without current calibration, neither flight efficiency nor long-term stability can be concluded.

The photovoltaic response was found to depend not only on pigment extraction efficiency but also on dye adsorption and interfacial charge-transfer processes. Although the overall efficiency remains modest compared with conventional sensitizers, the mullaca-based DSSCs exhibited measurable photon-to-current conversion consistent with their operation as natural-dye sensitized devices.

Two mullaca-sensitized DSSCs (C5, C6) also maintained a measurable electrical response during the NASA HASP stratospheric flight, providing preliminary evidence that this type of device can remain operational under low-pressure, low-temperature, and variable natural-illumination conditions. However, because the in-flight measurements were not performed under standardized irradiance conditions, direct comparison with laboratory photovoltaic parameters should be made with caution.

## Author contributions

Angie Pierina Perez Jara: conceptualization, investigation, methodology, validation, data curation, writing – original draft, and writing – review and editing; Michael Celestino: investigation, methodology, and data curation; Clemente Alfredo Luyo Caycho: resources and investigation; Jaime Claros: investigation and methodology; Yanett Huanca: investigation and methodology; Harry Anderson Rivera Tito: investigation; and María Esther Quintana Cáceda: conceptualization, formal analysis, funding acquisition, writing, and writing – review and editing.

## Conflicts of interest

The authors declare that they have no competing interests.

## Data Availability

The data supporting the findings of this study, including UV-vis, FTIR, XRD, SEM-EDS, photovoltaic, IPCE, and stratosphericflight data, are available in a shared Google Drive folder at the following link:https://drive.google.com/drive/folders/1uca_MdNd_jGHs_FuRoWWJrwpbqJtbVJW?usp=sharing. The data are publicly accessible through the link above and are available for the purposes of verification and reproducibility of the results reported in this article.

## References

[cit1] Adhikari A., Acosta D., Fragoso-Medina A. J., Subramaniam V. (2024). *et al.*, Review on the developments in copper indium gallium diselenide (CIGSe)-based thin film photovoltaic devices. J. Mater. Sci.: Mater. Electron..

[cit2] Andrés D. , Effect of the thickness of CdTe films obtained by sputtering on the efficiency of photovoltaic windows, Master’s thesis, Autonomous University of Querétaro, 2023

[cit3] Zhao C., Yu S., Tang W., Yuan X., Zhou H., Qi T., Zheng X., de Ning M., Ma M., Zhu J., Zhang J., Yang C., Li W. (2023). Advances in CIGS thin film solar cells with emphasis on the alkali element post-deposition treatment. Mater. Rep.: Energy.

[cit4] Zhou D., Zhou T., Tian Y., Zhu X., Tu Y. (2018). Perovskite-based solar cells: Materials, methods, and future perspectives. J. Nanomater..

[cit5] Anaya-González G. S., Rodríguez-Romero J. (2024). Three generations of solar cells: Alternatives for a more sustainable world. Renew. Sustain. Energy Trends.

[cit6] Bisquert J., Vikhrenko V. S., Mora-Seró I. (2009). Electron lifetime in dye-sensitized solar cells: Theory and interpretation of measurements. J. Phys. Chem. C.

[cit7] Joshi P., Zhang L., Davoux D., Zhu K., Cheng Y.-B. (2012). Reduced electron recombination of dye-sensitized solar cells based on TiO_2_ spheres consisting of ultrathin nanosheets with facet exposed. J. Mater. Chem..

[cit8] Parrino F. and Palmisano L., Titanium Dioxide (TiO_2_) and its Applications, Elsevier, 2020

[cit9] Peñas-Garzón M., Moreno-Medina I., Bedia J., Belver C. (2023). Synthesis and evaluation of TiO_2_/activated carbon photocatalysts: A multidisciplinary and cooperative approach for chemical engineering students. Educ. Quim..

[cit10] O'Regan B., Grätzel M. (1991). A low-cost, high-efficiency solar cell based on dye-sensitized colloidal TiO_2_ films. Nature.

[cit11] Mahajan U., Prajapat K., Dhonde M., Sahu K., Shinge P. M. (2024). Natural dyes for dye-sensitized solar cells (DSSCs): An overview of extraction, characterization and performance. Nano-Struct. Nano-Objects.

[cit12] Grätzel M. (2005). Mesoscopic solar cells for electricity and hydrogen production from sunlight. Chem. Lett..

[cit13] Cruz-Guerrero M. G., Romero-De La Cruz M. T., Martínez-Luevanos A. (2024). Semiconductor thin films based on TiO_2_ for solar cells. Cienc. Acierta.

[cit14] Velázquez Martínez S. , Development of TiO2 Solar Cells Sensitized with N-719 Ruthenium Dye and Their Scaling up to Photovoltaic Modules, Doctoral thesis, Autonomous University of the State of Morelos, 2018

[cit15] Bbumba S., Karume I., Kigozi M., Oyegc I., Ntale M. (2024). How components of dye-sensitized solar cells contribute to efficient solar energy capture. Asian J. Appl. Chem. Res..

[cit16] Flint H. V., Rivera Tito H. A., James R. D., Cucinotta F., Gibson E., Quintana Caceda M. E. (2024). Betanin dye extracted from ayrampo (Opuntia soehrensii) seeds to develop dye-sensitized solar cells. RSC Adv..

[cit17] Mahadik S. A., Pathan H. M., Salunke-Gawali S. (2024). An overview of metal complexes, metal-free and natural photosensitizers in dye-sensitized solar cells. ES Energy Environ..

[cit18] Dumbravă A., Georgescu A., Damache G., Badea C., Enache I., Oprea C., Gîrţu M. A. (2008). Dye-sensitized solar cells based on nanocrystalline TiO_2_ and natural pigments. J. Optoelectron. Adv. Mater..

[cit19] Jasim K. E. (2012). Natural dye-sensitized solar cell based on nanocrystalline
TiO_2_. Sains Malays..

[cit20] Zhou H., Wu L., Gao Y., Ma T. (2011). Dye-sensitized solar cells using 20 natural dyes as sensitizers. J. Photochem. Photobiol., A.

[cit21] Ali R. A. M., Nayan N. (2010). Fabrication and analysis of dye sensitized solar cells using natural dye extracted from dragon fruit. Int. J. Integr. Eng..

[cit22] Safie S., Mat A. B., Ahmad Z. (2017). Electron transport studies of dye-sensitized solar cells based on natural sensitizer. BioResources.

[cit23] Sharma K., Sharma V., Sharma S. S. (2018). Dye-sensitized solar cells: Fundamentals and current status. Nanoscale Res. Lett..

[cit24] Dutta A., Nayak M., Akhtar A. J., Saha S. (2025). Enhancing photovoltaic efficiency of ZnO based DSSCs through metal ion doping: A comparative simulation study. J. Phys. Chem. Solids.

[cit25] Supriyanto E., Nawafil M., Subekti A. (2024). Optimizing dye-sensitized solar cell efficiency through TiO_2_/CuS doping: Effects of internal parameter variations. East. -Eur. J. Enterp. Technol..

[cit26] Banik A., Ansari M. S., Qureshi M. (2018). Efficient energy harvesting in SnO_2_-based dye-sensitized solar cells utilizing nano-amassed mesoporous zinc oxide hollow microspheres as synergy boosters. ACS Omega.

[cit27] Ramadhani D. A. K., Sholeha N., Khusna N. N., Diantoro M., Afandi A. N., Osman Z., Pujiarti H. (2024). Ag-doped TiO_2_ as photoanode for high performance dye sensitized solar cells. Mater. Sci. Energy Technol..

[cit28] Espinoza Duarte III A. , Design, fabrication, and analysis of Grätzel cells using (TiO_2_ and ZnO):Mg as photoanodes, Doctoral thesis, National Institute of Astrophysics, Optics and Electronics, 2024

[cit29] Shi S., Li K., Peng J., Li J., Luo L., Liu M., Chen Y., Xiang Z., Xiong P., Liu L., Cai W. (2022). Chemical characterization of Kadsura coccinea leaf extracts by UHPLC-Q-Exactive Orbitrap mass spectrometry, with assessment of antioxidant and anti-inflammatory activities. Biomed. Pharmacother..

[cit30] Li Y., Lei J., Qin X., Li G., Zhou Q., Yang Z. (2023). A mitochondria-targeted dual-response sensor for monitoring viscosity and peroxynitrite in living cells with distinct fluorescence signals. Bioorg. Chem..

[cit31] Zhang H., Liu Z., Dai B., Yang Q., Liang S., Song X., Zhang X. (2026). Synthesis of a novel pH-responsive azo dye and its application on cotton fabrics for rapid visual sensing. Dyes Pigm..

[cit32] Wang J., Sun H., Mou H., Yang S. (2024). Protein and lysine improvement harnessed by a signal chain of red light-emitting diode light in Chlorella pyrenoidosa. Bioresour. Technol..

[cit33] Li H., Zhang J., Zhou J., Zang J., Shi G., Xu J., Cai X. (2026). An isolated unidirectional DC transformer with active current reshaping for the photovoltaic plant DC collection system. IEEE Trans. Ind. Electron..

[cit34] Cacciuttolo C., Guardia X., Villicaña E. (2024). Implementation of renewable energy from solar photovoltaic (PV) facilities in Peru: A promising sustainable future. Sustainability.

[cit35] Rodrigues M. A. (2024). Anthocyanin expressed in leaves boosting chlorophyll-based dye-sensitized solar cells performance. Next Res..

[cit36] Cerda Rojas B. N. , Characterization of natural dyes for use in dye-sensitized solar cells, Bachelor’s thesis, University of Concepción, 2014

[cit37] Huamán A. , Theoretical and Experimental Study of Solar Cells Based on Nanostructured TiO_2_ Films Sensitized with Natural Dyes Extracted from Zea mays and Bixa Orellana, Doctoral thesis, National University of Engineering, 2021 10.1039/d1ra01043c PMC8695372 35423450

[cit38] Rengifo Salgado E., Vargas Arana G. (2013). *Physalis angulata* L. (Bolsa Mullaca): Review of its traditional uses, chemistry, and pharmacology. Bol. Latinoam. Caribe Plant. Med. Aromat..

[cit39] Nazario Ticse R. , TiO_2_ Grätzel Cells Sensitized with the Natural Dye Ayrampo (Opuntia Soehrensii), Using Graphite as a Counter Electrode, Master’s thesis, National University of Engineering, 2016

[cit40] Huamán A. A., Celestino M. R., Quintana M. E. (2021). Theoretical and experimental study of solar cells based on nanostructured films of TiO_2_ sensitized with natural dyes extracted from Zea mays and Bixa orellana. RSC Adv..

[cit41] Zahid S., Rasool A., Ans M., Yaseen M., Iqbal J. (2021). Quantum chemical approach of donor–π–acceptor based arylborane–arylamine macrocycles with outstanding photovoltaic properties toward high-performance organic solar cells. Energy Fuels.

[cit42] Janjua M. R. S. A. (2023). Impact of symmetry breaking on the performance of non-fullerene acceptors (NFAs) for photo and thermally stable organic solar cells (OSCs): A DFT-based interrogation and investigation. J. Photochem. Photobiol., A.

[cit43] Umar A., Yaqoob J., Khan M. U., Hussain R., Alhadhrami A., Almalki A. S. A., Janjua M. R. S. A. (2022). Doping of superalkali and superhalogen on graphene quantum dot surfaces to enhance nonlinear optical response: An efficient strategy for fabricating novel electro-optical materials. J. Phys. Chem. Solids.

[cit44] Janjua M. R. S. A. (2022). Quantum chemical design and prediction that complements understanding: How do the transition metals enhance the CO_2_ sensing ability of inorganic Mg_12_O_12_ nanoclusters?. J. Phys. Chem. Solids.

[cit45] Janjua M. R. S. A., Guan W., Yan L., Su Z.-M., Karim A., Akbar J. (2010). Quantum chemical design for enhanced second-order NLO response of terpyridine-substituted hexamolybdates. Eur. J. Inorg. Chem..

[cit46] Martineau D. , Dye Solar Cells for Real – the Assembly Guide for Making Your Own Solar Cells, Solaronix, 2011

[cit47] Perez A. , Optoelectronic characterization of nanostructured solar cells sensitized with mullaca and their application in sensors, Undergraduate thesis, National University of Engineering, 2025

[cit48] Diantoro M. , Albadi’ah I. V. and Nasikhudin, The effect of TiO_2_ film immersion duration in N719 dye on microstructure, optical properties, and photoanode performance of photosupercapacitors based on FTO/TiO_2_/N719/activated carbon/carbon black, in Proc. Int. Conf. Sci. Technol., Atlantis Press, 2023, pp. 143–156

[cit49] Wintermans J. F. G. M., De Mots A. (1965). Spectrophotometric characteristics of chlorophylls a and b and their pheophytins in ethanol. Biochim. Biophys. Acta, Biophys. Incl. Photosynth..

[cit50] Porra R. J., Thompson W. A., Kriedemann P. E. (1989). Determination of accurate extinction coefficients and simultaneous equations for assaying chlorophylls a and b extracted with four different solvents: Verification of the concentration of chlorophyll standards by atomic absorption spectroscopy. Biochim. Biophys. Acta, Bioenerg..

[cit51] Al-Amin K., Kawsar M., Mamun M. T. R. B., Hossain M. S. (2025). Fourier transform infrared spectroscopic technique for analysis of inorganic materials: A review. Nanoscale Adv..

[cit52] Hirbodi S., Jamekhorshid A., Jalali T., Mohammadian J., Osfouri S. (2024). Enhanced efficiency, electron lifetime, and eco-friendliness of dye-sensitized solar cells using nanofibrous TiO_2_/ZnO composite photoanodes and natural anthocyanin sensitizer. Results Surf. Interfaces.

[cit53] Iman R. N., Harrabi K., Younas M., Mekki A. (2024). Fabrication of efficient natural dye-sensitized solar cells using Mediterranean olive leaves as natural dye sensitizer. J. Photochem. Photobiol., A.

[cit54] Chasquibol S. N. (2010). Application of Fourier transform infrared spectroscopy in the qualitative determination of the degree of methyl esterification in pectins. Rev. peru. quím. ing. quím..

[cit55] Flores-Carrasco G., Alcántara-Iniesta S., Sierra-Fernández A., Gómez-Villalba L. S., Rabanal M. E., Milosevic O. (2014). Structural, optical, and electrical properties of SnO_2_ and SnO_2_:F films deposited by ultrasonic spray pyrolysis. Superf. Vacio.

[cit56] Ceh Cih F. J. , Modulation of the properties of Mg-doped SnO_2_ thin films with application in MTO/CdTe solar cells fabricated entirely by sputtering, Doctoral thesis, Center for Research and Advanced Studies of the National Polytechnic Institute (CINVESTAV), 2024

[cit57] García Martínez A. , Solar Cells Sensitized by Chlorophyll Derived from Spearmint (Mentha Spicata), Master’s thesis, Autonomous University of Zacatecas, 2019

[cit58] Núñez Salinas A. I. , Synthesis and Characterization of Dye-Sensitized Solar Cells (DSSC) Using Graphene Sheets and Their Derivatives, Undergraduate thesis, University of Santiago de Chile, 2017

[cit59] Lei H., Bao N., Ben-Dor E., Peng S., Zhao N. (2026). Soil bidirectional reflectance characteristics and applications in quantitative soil property retrieval: A systematic review. Catena.

[cit60] Li Q., Hang Q., Ruan Y. (2022). Evaluation of modified peanut shell in the removal of Cr(VI) from aqueous solution. Desalin. Water Treat..

[cit61] He G., Li Y., Yin D., Yin D., Yang S., Rao Z., Sun Y., Liu R., Luo X. (2026). Sequential adsorption enabled by dye-saturated biogenic vaterite: A waste-to-material strategy for enhanced methylene blue removal. J. Hazard. Mater..

[cit62] Wang Q., Zhang L., Liu X., Li S. (2021). Two-dimensional semiconductor heterojunctions for optoelectronics and electronics. Front. Energy Res..

[cit63] Hang S., Fang X., Yang Z., Wang X., Wang Y., Wu D., Dai G., Lin X. (2026). Localized DBR-based transparent green micro-LEDs on polished sapphire. IEEE Photonics Technol. Lett..

[cit64] Zhang Q., Li G. (2024). *et al.*, Recent advances in integrated solar cell/supercapacitor devices: Fabrication, strategy and perspectives. J. Adv. Res..

